# Complete Genome Sequence of *Enterococcus* Bacteriophage EFLK1

**DOI:** 10.1128/genomeA.01308-15

**Published:** 2015-11-19

**Authors:** Leron Khalifa, Shunit Coppenhagen-Glazer, Mor Shlezinger, Miriam Kott-Gutkowski, Omri Adini, Nurit Beyth, Ronen Hazan

**Affiliations:** a Faculty of Dental Sciences, Hebrew University-Hadassah School of Dental Medicine, Jerusalem, Israel; b Department of Prosthodontics, Hebrew University-Hadassah School of Dental Medicine, Jerusalem, Israel; c Interdepartmental Core Unit of the Hebrew University, Campus Hadassah, Jerusalem, Israel

## Abstract

We previously isolated EFDG1, a lytic phage against enterococci for therapeutic use. Nevertheless, EFDG1-resistant bacterial strains (EFDG1^r^) have evolved. EFLK1, a new highly effective phage against EFDG1^r^ strains, was isolated in this study. The genome of EFLK1 was fully sequenced, analyzed, and deposited in GenBank.

## GENOME ANNOUNCEMENT

Although *Enterococcus faecalis* is a commensal Gram-positive microorganism inhabiting the gastrointestinal tract, it can cause highly fatal forms of infection (1, 2). Numerous existing potent antibiotics against enterococcal infections are still insufficient to preclude morbidity and mortality (3, 4). The most problematic are antibiotic-resistant strains in biofilms (5, 6), mainly the vancomycin-resistant enterococci (VRE), declared by the Centers for Disease Control and Prevention (CDC) as “extremely difficult organisms to treat and a major public health threat” (http://www.cdc.gov/HAI/organisms/organisms.html). One promising solution that may reconcile the deficiencies associated with current antibiotic treatments is phage therapy (7, 8). Previously, we isolated EFDG1, a lytic phage for therapeutic use that efficiently killed *E. faecalis* (9). Nevertheless, bacterial strains resistant to EFDG1 (EFDG1^r^) evolved. To combat EFDG1^r^ strains_,_ we isolated a new phage termed EFLK1, which was able to eradicate the resistant strain. EFLK1 was isolated from sewage effluents, and its DNA was purified, sequenced, and analyzed, as previously described for EFDG1 (9). Briefly, the phage was purified using the top agar method and stored at 4°C without chloroform. DNA was purified using the Phage DNA isolation kit (Norgen Biotek). The Illumina Nextera XT DNA kit (San Diego, CA) was used to prepare libraries. DNA amplification was carried out by a limited-cycle PCR, and the amplified DNA was purified using AMPure XP beads. The DNA libraries were normalized, pooled, and tagged in a common flow cell at 2 × 250-bp paired-end reads, followed by sequencing using the Illumina MiSeq platform. The quality of the reads was assessed by FastQC (http://www.bioinformatics.bbsrc.ac.uk/projects/fastqc). The *de novo* assembly with end trimmed reads was performed using Geneious 8.1 (Biomatters). The genome was assembled from 25,561 short reads, with an average coverage of 30 bases per base. Analysis of the open reading frames (ORFs), annotation, and phylogenetic tree generation were also performed by Geneious 8.1 and its plugins. tRNAs were predicted using tRNAscan-SE version 1.21 (http://lowelab.ucsc.edu/tRNAscan-SE/).

The EFLK1 phage contains a circular genome of 130,952 bp, with 209 putative coding sequences and a G+C content of 35.9%. No tRNA genes were identified in EFLK1, in contrast to EFDG1, which harbors 23 genes. According to our analysis, EFLK1 belongs to the *Spounavirinae* subfamily of the *Myoviridae* phage family. As such, EFLK1 shows similarities with the other *E. faecalis Spounavirinae* phages, EFDG1 (accession no. KP339049.1 [9]), PhiEF24c (accession no. AP009390.1 [10]) and ECP3 (accession no. KJ801817.1). EFLK1 contains a significant cluster of DNA replication components, including two DNA polymerases (EFLK1_ORF130 and EFLK1_ORF142), two DNA helicases (EFLK1_ORF161 and EFLK1_ORF159), DNA maturase A (EFLK1_ORF121), three DNA exonucleases (EFLK1_ORF127, EFLK1_ORF157, and EFLK1_ORF158), resolvase (EFLK1_ORF147), and primase (EFLK1_ORF155).

Additionally, with regard to transcription, EFLK1 contains a gene for RNA polymerase (EFLK1_ORF176) and a sigma factor (EFLK1_ORF134), which are conserved among many *Spounavirinae* phages, including the 3 *E. faecalis* strains and other Gram-positive bacteria.

### Nucleotide sequence accession number.

The full-genome sequence of phage EFLK1 has been deposited in GenBank under the accession no. KR049063.
